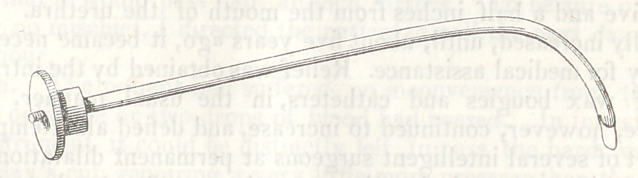# On Strictures of the Urethra

**Published:** 1847-12

**Authors:** James Bryan

**Affiliations:** Lecturer on Surgery, formerly Professor of Surgery in Castleton, Vermont, late Surgeon and Physician to the Philadelphia Dispensary, Member of the Philadelphia Medical Society, &c. &c.


*On Strictures of the Urethra.* By JAMES BRYAN, M.D., Lecturer on Surgery, formerly Professor of Surgery in Castleton, Vermont, late Surgeon and Physician to the Philadelphia Dispensary, Member of the Philadelphia Medical Society, &c. &c.

In the whole range of surgical disease, there is unquestionably no one more inconvenient, distressing and obstinate, than "Stricture of the Urethra." Confined, as it is, to no age,—for according to the best authorities, it is found in youth and childhood,—continuing, as it generally does, during the whole of life after an attack, liable to occur at any moment, and placing in jeopardy the very life of the individual—it has ever been a subject of deep interest to the surgeon.

Unlike many other serious and persistent diseases, it is, unfortunately, one of very common occurrence. If, as Dr. Hunter says, "nearly every man has had gonorrhœa," so we may say, a large per centage, of those who have been thus afflicted, have obstructions to the free passage of the urine, in some form or other. Strictures of the urethra may be classified as follows: 1st, Spasmodic stricture. 2d, Permanent stricture. 3d, A combination of the first two.

*Spasmodic Stricture.*

Spasmodic stricture is a a simple closure of a portion of the urethral canal, by the contraction of some of the muscular fibres which surround it. This spasm may be in the circular fibres of the urethra itself; the compressores urinæ, or other portions of the muscular tissue, acting upon the passage. It is induced by some irritation, either local or general, either in the urethra itself, or acting upon some distant part, and producing the disease by sympathy. Like other diseases of this nature, it is found most frequently in persons of a nervous and excitable temperament. The irritation of gonorrhœa, in any or all of its stages, produces this form of stricture; it is not of course in this, or in purely sympathetic cases, dependent upon organic changes, in the form of deposits, for its existence. Although very distressing for the time being, producing for hours together complete suppression of the urinary discharge, yet it must be considered as a temporary disease. The application of cold water to the parts and cold to the feet, as recommended by Dr. Hall—or the reverse, warm baths, pediluvia and semicupia—are means which very frequently succeed in relaxing the stricture. In addition to these, the introduction of a catheter or sound, the use of opiate enemata, or, according to Dr. Hunter, "the crumb part of a new baked loaf, warm from the oven, applied to the perineum;" a blister to the groin; hot steam to the scrotum and perineum, &c. &c. Of all these, the best by far and most certain, as well as the most expeditious, is the introduction of the catheter, previously warmed and oiled for the purpose. I have found it advantageous, in cold weather, either to allow the patient to remain in bed, or be in a warm room during the introduction of this instrument.

In some cases the disease assumes the character of periodicity, and I have had to evacuate the bladder at stated periods for several days, before the disease gave way, and this entirely independent of that form of stricture which sometimes takes place during the cold stage of an intermittent fever. When caused by some local irritation, the final cure of the disease must of course be sought by the removal of the cause. The old adage need scarcely be repeated, which says that "the removal of the cause cures the effect."

*Permanent Stricture.*

"A permanent stricture" (says Sir Everard Home) "is that contraction of the canal which takes place in consequence of coagulable lymph being exuded between the fasciculi of muscular fibres, and *upon* the internal membrane, in different quantities, according to circumstances; and, in the same proportion, diminishing the passage for the urine at that part, or completely closing it up."

This form of the disease is the result of inflammatory action in the tissues around the urethra, producing a deposit of coagulable lymph, which, by its bulk, infringes upon the diameter of the passage. As a general rule, the deposit is slow in its progress, and requires a considerable time to attain sufficient size to materially interfere with the passage of the urine. The symptoms consequently develope themselves gradually. Most generally, the origin, according to Sir A. Cooper, may be distinctly traced to one or more attacks of gonorrhœa, and this agrees with our experience. At the same time wounds of the parts, particularly contused wounds—a transfer of inflammatory action from neighbouring parts—are known to produce the same effects. The character which these deposits assume, (and they do *not involve* the lining membrane *of the urethra*, which, on the contrary, is corrugated in folds in the passage, over the uneven surface,) is that of a hard cartilaginous ring, partial or complete. The extent of the deposit, which may be felt through the parietes, as a hard substance along the urethra, varies considerably. According to Hunter and others, it is generally not greater than that represented by a *pack thread*, tied around the canal. Sir B. Brodie speaks of its being sometimes an inch or an inch and a half in extent; and in a case which was treated by myself, it extended more than two inches along the passage, and could be felt externally by the patient and surgeon with the finger. Mr. Samuel Cooper, in his Surgical Dictionary, mentions a case of fistula where the entire urethra beyond, was closed. The number of strictures also varies in the same case; in many cases there is but one; in others there are two, three, or more strictures. As the diameter of the passage varies in different parts of its course, so the *seat* of stricture varies. Where the diameter is diminished or curved, the predisposition to stricture seems to be greater than in other parts. Hence the curve of the canal and the *bulb*, as it is called, are very commonly seats of the disease. In my experience, I have found that the most common seat is about 5½ inches from the external orifice; next to this, about 7 or 7½ inches; and lastly, about 3½ or 4 inches from the extremity of the penis. I have also seen them just within the orifice, and so small that a fine violin string of catgut, or small straws, were used by the patient to keep them open for urination.

*Symptoms.*

The first symptom of stricture is the obstruction, more or less complete, which it causes to the free passage of the urine. The stream will be changed in shape, forked, turned from its usual course, or fall perpendicularly from the mouth of the urethra in great drops and scattering. Some few drops will be retained in the passage, which will pass off after the rest, producing considerable inconvenience. The patient sometimes has to get up at night two or three times to evacuate his bladder, which is done with difficulty and with considerable effort. A weight and uneasiness is felt in the region of the perineum, and not unfrequently a gleety discharge issues from the urethra, which may be mistaken for gleet itself. The stream of urine gradually diminishes, inducing strong, "straining" efforts to accelerate it, until almost no stream passes, and the urine falls in drops or stops entirely. The latter, however, generally occurs after exposure to cold or other causes of inflammation, which, setting in, in the strictured part, closes the passage entirely;—or the closure may be induced by the spasmodic action of the muscles, as we shall see when speaking of the combined form of the disease. From a few weeks to eight or ten or more years, may, however, pass in the progress of the disease, ere this state of things takes place. One case lately treated by me, resulted from a very severe attack of gonorrhœa, contracted nine years before. The progress of the disease was gradual, until relieved by an operation. In another case, also cured by an operation, rather more than three years had elapsed. In a third case, about one year, with repeated gonorrhœas, had induced a confirmed stricture.

In the early stages there is commonly very little pain or inconvenience; the impediment to free urination, which the patient may refer to any other cause than the right one, being the only symptom. In some cases considerable pain accompanies the disease. A patient now sitting by me says, to the question in reference to pain, that he never had any, but adds, that "now and then" he would feel pain in the vicinity of the anus, apparently rheumatic, which would wake him up at night, and would be relieved by pressure. The pain existed particularly when he was costive. Never suspected a stricture; the stream, he says, gradually diminished, but was variable. For a few days the obstruction would be considerable, and then for a month or two, it would be as usual, never, however, returning to the natural size of the stream. These paroxysms gradually became more frequent, the urine "dribbling" away with difficulty, until at length it entirely ceased, and he was forced to apply for immediate relief. The difficulty in urinating not only varied on different days, but at different times in the same day. An attempt in the morning would sometimes be a partial failure; an hour or two after, the attempt would be more successful. It affected his mind, and was itself affected by mental emotions. The latter, particularly anxiety of mind, increased the difficulty, while the appetite and spirits were affected by the disease. This, in fact, is known to be the case in most of the diseases involving the urinary or genital organs. One patient tells me that the venereal appetite in him, although of a pretty warm temperament, has been for years merely passive. The testicle, one or both, it is well known, also, sometimes become enlarged and painful, which may be mistaken for a very different disease. Whether the disease affects the procreative function or not, I am not able to say; but two gentlemen, both married men of four or five years standing, have neither of them children, and they attribute the fact to the disease.

*Mixed Stricture.*

The mixed form of the disease is one exceedingly common. The local irritation of the sub-inflammation, which no doubt exists in the stricture deposit, is sufficient to excite the spasmodic action of the muscular fibres, longitudinal or other, especially in nervous individuals. And these spasms are frequent sources of great pain and considerable danger. They no doubt react upon the strictured part, and assist in keeping up that amount of irritation which is essential to the progress of the disease. In these cases we have all the symptoms of simple spasmodic stricture, made more or less permanent by the permanent deposits. The other and more general symptoms are all more intense from the general susceptibility of the system.

*Consequences.*

The consequences of long continued obstruction and stricture on the bladder and neighbouring organs are various. The bladder becomes, from the increased action, thickened and contracted, so that it sometimes will contain not more than one-fourth of the normal amount of urine. Hence the frequent disposition to evacuate this viscus. The mechanical obstruction, continuing for a certain length of time, will induce ulceration of the urethra, opening through the perineum; and we may have one or more fistulæ in perineo. This takes place sometimes in a short time, from acute inflammation setting in, establishing the suppurating process, which, in most cases, opens through the perineum. In other cases, when relief to the permanent obstruction of the passage is not obtained speedily, we may have rupture of the urethra or bladder, by which the urine is infiltrated into the neighbouring cellular tissue, and mortification and gangrene of the parts follow. Even death may be the consequence of this accident. We are of the opinion, however, that this result, particularly the phlegmonous inflammation opening in the perineum, can be avoided in most cases by the judicious surgeon. Some of our best writers speak of this as a matter of common occurrence. Certainly when it depends on the mechanical obstruction of a stricture, it ought to be avoided; the passage being easily opened by a proper instrument for that purpose. The enlargement of the ureters and pelvis of the kidneys, with more or less disease of the latter, are consequences of long continued obstruction of any kind in the urinary passages.

*Treatment of Stricture.*

The treatment necessarily divides itself into that which is appropriate to spasmodic, permanent, or the mixed form of the disease. In reference to the first, where it is simple and uncomplicated, the methods stated under the caption, "spasmodic stricture," will be found generally successful; being merely symptomatic or nervous. The modes of treatment there mentioned, particularly for immediate relief, the introduction of a moderate sized, smooth, warm, well oiled metallic sonnd, will be found effectual. The use of terebinthinates, opiates, or anti-spasmodics may be proper for the purpose of permanently curing the disease. Where some irritation, either in the organs of digestion, on the general surface, (such as a blister, for instance) or in the bladder, produces the disease, the removal of the irritation will result in the cure of the stricture.

*Treatment of Permanent Stricture.*

This is divided by most authors into dilatation, cauterization and scarifications, followed by dilatation. The invention of the bougie and flexible catheter was considered by John Hunter as a very great improvement, in the treatment of strictures and other diseases of the urinary organs. And from that time to the present, they have been extensively used, not to say abused, in the treatment of strictures. Every tyro thinks he must try his skill in introducing this foreign body into the urethral passage. Precise instructions are given as to the mode of operating to effect this purpose, almost all of which, it is acknowledged, fail to ensure success. The curves of the canal—its unequal diameters—the fold and lacunæ of the lining membrane—the spasmodic action of the longitudinal and circular fibres—the contractions of the sphincter vesicæ, enlargements of the so-called third lobe and lateral lobes of the prostate gland, in addition to the very obvious one of permanent stricture, or morbid growth in the passage—are all so many difficulties which frighten the novice in his timid attempts at the introduction of a proper instrument. Our predecessors do not, however, seem to have been so timid; but, from their own experience, no doubt, caution us against using too much force, and either bruising the parts or producing an "artificial passage," and not always in the right direction, even the rectum having been entered, according to some writers. In these cases it is evident that a very unwarrantable amount of force must have been injudiciously applied. The very irregular course of the passage when several strictures exist, makes it necessary to be careful, and to *humour* the parts during the introduction of the instrument. As a general rule, we agree with the distinguished teacher, Professor W. Gibson, that a metallic sound, of proper or moderate dimensions, warm, and well oiled, is the easiest introduced: metallic catheters, not too large, come next. The passage seems to become straight, or rather the inequalities yield better, to these instruments, particularly when they fill well the diameter of the canal. I have seen a spasmodic stricture overcome in this way when all others had failed.

The process of dilatation presupposes of course the introduction of some dilating body—which shall press outwards the parietes of the stricture or strictures. Where the contraction is considerable, a very small bougie is gradually introduced and allowed to remain, sometimes for several hours. After this, one a size larger, say the next day or shortly after, this to be followed by one still larger, until the diameter is increased to the natural size. Dr. Physick, we have understood, was accustomed to require his patient to obtain for himself a dozen bougies and catheters, of assorted sizes; from the smallest up to one almost twice as large as the natural size of the canal. Dilatation was carried to the extent of enlarging the urethra to a very considerable extent beyond the usual size. This was with the view of preventing too sudden contraction in the strictured part afterwards—which, however, was almost sure to take place, particularly in the cartilaginous forms of the disease where Sir B. Brodie says the dilation is "so important."

There are we believe, at least two forms of permanent strictures, perhaps three, in which something like a radical cure may be expected from the introduction of the metallic dilator. These are: firstly; the thread-like stricture, which divides the passage in the form of a thin diaphragm, and is torn by the instrument in an attempt to part it. The hemorrhage which follows, indicates the rupture of some small vessels. We have seen a number of cases where this appeared to be the case; and the cures were at once complete and permanent. Secondly, when soft, fleshy or polypic growths obstruct the passage, as they do those of the ears, rectum or uterus, these bodies being of a soft friable nature, easily give way, and are destroyed by the solid instrument. Thirdly; in the case of simple bands, as explained by Sir A. Cooper, when these bands are not too strong and resistent; they are perforated, and broken up by the means taken to dilate the urethra.

With regard to the use of the gum catheters, in strictures, Sir B. Brodie, after decribing the mode of introduction, &c.—points out the cases to which he conceives them to be applicable, as follows:—"1stly. When time is of much value, and it is of great consequence to the patient to obtain a cure as soon possible.

2dly. When a stricture is grisly and cartilaginous, and therefore not readily dilated by the ordinary methods.

3dly. Where from long continuance of the disease the urethra has become irregular in shape; or where a false passage has been made by previous mismanagement. Under these circumstances, if you can succeed in introducing a gum catheter, and let it remain for a few days in the bladder, you will find your difficulties at an end; the irregularities will disappear, and the false passages will heal.

4thly. Where a severe rigor follows each introduction of the bougie. This disposition to rigor is such, that it is impossible to proceed with the treatment in the ordinary way. Observe, in these cases, when the rigor takes places, it seldom follows the use of the bougie immediately. It almost always occurs soon after the patient has voided his urine, and seems to arise, not as the immediate effect of the operation, but in consequence of the urine flowing through the part which the urine has dilated. Now, if instead of a bougie, you use a gum catheter, and allow it to remain, the urine flowing through the catheter, the contact of it with the urethra is prevented, and the rigor is prevented also."—*Braithwaite.*

We beg leave to differ from the Baronet in reference to the two first cases mentioned. We do not consider that the treatment with the gum catheter *is* the most speedy in *all* cases. Nay, there is a class of cases in which the gum catheter is of no more effect than any other instrument *which cannot* be *introduced.* In the three cases stated above, where a cure is the result of a destruction of the parts, it may be an expeditious method; but even in those, the metallic sound or catheter is far better, and more certain. In the second case, or that in which the "stricture is grisly and cartilaginous," it is notoriously a very uncertain instrument as far as a cure is concerned. So much so, that cases go on daily, in spite of all attempts at dilation with the gum catheter, until the passage in portions of its course is well nigh and even entirely obliterated. In both these cases, *we* should be disposed, particularly in the latter, to counsel very different treatment. But we must proceed with the treatment.

The objects to be attained by the forcible entry of a bougie or sound or fluid, are, according to the best authorities: 1st. A free passage, at least temporarily, for the urine. 2d. Such an amount of dilatation as will relieve the patient from the immediate effects of the stricture. 3d. Ulceration of the strictured part, which will result in the destruction of the morbid deposit, and in this way leave the passage clear. 4th. It is supposed that dilatation, carried to a certain extent, will, without producing ulceration, induce such an action in the parts as will result in their absorption; and in this way cure the disease. Precise directions are given as to the amount of pressure, and time of its continuance, necessary to the accomplishment of these objects, without producing the much dreaded and really to be feared, artificial passage, with all its terrible consequences. The materials used for these purposes are, 1st. Wax or plaster cloth bougies. 2d. Gum elastic do. 3d. Catgut or gelatinous bougies; these of course for the smaller strictures. 4th. Bougies made of the bark of the American elm. 5th. Bougies of ivory softened. 6th. Metallic bougies either flexible or inflexible. 7th. Water or some other bland fluid *forcibly* injected, so as to induce an expansion of the stricture.

With respect to the first object to be obtained by the use of these instruments—we prefer decidedly the metallic inflexible sounds, or catheters, and next to these the wax bougies.

The second object may in our opinion be better and more certainly obtained, by well polished metallic instruments, *always* used with the greatest care, and in most cases *adding very* little more force than the weight of the instrument itself. We prefer introducing the instrument in the old way, viz. with its convex side upwards until the point reaches the curve, then *gradually* performing a semi-circular curve, without, however, allowing the instrument to stop in its course. The cito, in judicious hands, is the most likely to be the tuto and jucunde, in this neat little operation. Next to the metallic, the wax bougie armed with a good stilet, bent to the proper curve. Experience, however, proves that the relief is but temporary, in a great majority of the cases treated in this way.

The third object to be obtained, viz. ulceration, or such an amount of irritation as will induce a discharge, is recommended by Dr. Hunter, and many of the best surgeons up to the present day. The instrument is to be carefully introduced to the stricture and the extremity allowed to come in contact with the indurated parts. Gradual but firm pressure is to be applied for from five minutes to fifteen or twenty, and this to be repeated from day to day until the stricture begins to yield, and the instrument makes progress in the canal. Very great caution is necessary, in adjusting the instrument and applying the requisite degree of force. It is in this practice, that so many false passages are made—even through the substances of the prostate gland. Our view of this mode of treatment, in the cases where it is generally recommended, is that it is *bad* practice, and should not be resorted to in the present day. It appears to us to be altogether behind the age. The invention of the wax bougie and catheter was a great improvement in the days of Hunter and Pott, and certainly some improvements have been added since. These rough and uncouth processes should be placed along with many others, which have been entirely abandoned in modern days.

The fourth object, namely, the production of such an action in the parts as will induce absorption without ulceration, is too uncertain, except in the three cases mentioned, to be relied upon in a disease of so serious a character as permanent stricture.

One word in reference to the *forced* injections of fluids as a means of dilatation, and we have finished this part of our subject. A good epitome of the matter is given by Velpeau in the following words: "Trye, qui en a parlé le premier en 1784, dit en avoir retiré les plus grands avantages, et Sœmering avance que si le plus fine bougie ne peut pas franchir le rétrécissement, il injecte de l'huile dans le canal, dont il ferme aussitôt l'orifice, et qu'il presse ensuite d'avant en arrière pour faire marcher le liquide. La mèthode de Bruninghausen est un peu differente : au moment où le malade veut uriner, il comprime l'urètre avec force derrière le gland, force le fluide à rétrograder, et croit détruire ainsi le retrécissement. En 1822 M. Despiney de Bourg a proposé un liquide purement émollient, poussé avec une seringue. M. Citadini qui a publié un travail sur ce sujet, conduit une sonde ouverte jusqu'à l'obstacle, tient l'urètre solidement appliqué sur elle, et s'en sert comme d'un siphon pour injecter avec toute la force nécessaire de l'eau tiède, ou tout autre liquide approprié, dans le canal. M. Amussat, qui s'est cru l'inventeur des injections forcées, se comporte a peu près comme M. Citadini. It veut qu'on applique une compresse autour de la verge, pour qu'il ne reste aucun vide entre la sonde et les parois du canal, puis qu'on adapte une bouteille de caoutchouc remplie d'eau au pavillon de cette sonde, et qu'on pousse l'injection en comprimant la poche élastique avec un tourniquet : mais on sent bien que, le principe étant posé, il importe peu que le liquide soit projete par l'intermède d'une seringue, d'une soche en gomme élastique, du doigt, ou de tout autre manière."

The application of caustic to strictures was practised by Dr. Hunter, and has maintained its position in surgical practice ever since. Hunter's plan of application, was a very simple one. The end of a bougie was removed; the caustic (nitrate of silver) introduced, fastened with a piece of wax; and, the distance to the stricture having been previously measured, the caustic bougie was carefully and quickly introduced, and maintained for a moment in contact with the obstruction. This was repeated from time to time, as the case demanded, or the parts would permit; until a moderate sized instrument could be introduced. Various *portes caustiques* have been invented, for the purpose of more effectually applying this medicament. That of Ducamp was designed to enter the stricture and cauterize from within outwards. The instrument of Lallemand was designed to pass the stricture and after projecting the caustic from a lateral opening in the silver catheter, the instrument was withdrawn, and, thus made to act upon the stricture, from *behind forwards.* These two processes and their modifications necessarily involve the passage of the strictured part. The treatment by caustic has found favour among the English and American surgeons, particularly the former. The French, however, as is common where the practice is popular on the other side of the channel, have never heartily adopted cauterization as a means of curing stricture. Sir E. Home, Sir A. Cooper, Sir B. Brodie, and a host of other distinguished British and continental surgeons have given their testimony in opposition to the use of caustic in stricture. It is probable that when it is at all proper, the simple plan of Hunter as improved by Sir E. Home, will be found the most effectual as well as most practical. Sir B. Brodie gives his objections to the use of caustic for the following reasons.

1st. Although the caustic often relieves spasm, it also very often induces it. It is true that in many instances it enables a patient to make water with more facility; but in many instances, also, *it brings on retention of urine.* 2dly. Hæmorrhage is a more frequent consequence of the use of the caustic, than the common bougie, and it sometimes takes place to a very great and to an almost dangerous extent. 3dly. Where there is a disposition to rigor, the application of caustic induces rigors where there had been no manifest disposition to them previously. 4thly. Unless used with caution, the application of caustic may induce inflammation of the parts situated behind the stricture, terminating in the formation of abscess. There are strong arguments against its use, and yet this practice, next to dilatation, is the most generally adopted of any. No writer of eminence speaks upon the subject, however, without shedding some crocodile tears over the bad effects of caustic and dilatating instruments.

Scarification consists in light incisions made from within the stricture either outwards the instrument having perforated the strictured space, or from behind forwards, by means of a lancet which is made to project from the side of the catheter which contains it and has been introduced beyond the stricture. By pressing upon the stilet projecting beyond the proximal extremity of the *inflexible* catheter, the lancet is made to rise above the surface of the instrument, through a slit—either one side or the other—sometimes on the lower side, when the whole instrument is withdrawn beyond the stricture. In this way, the sharp edge of the blade, comes in contact with the strictured or *any other* part.

"Three instruments have been devised by Amussat, the peculiarity of which consists in their cutting upon a sliding oval button which is made to *hook behind the stricture.* 1. One which is called an urethratome, consisting of a conical steel cylinder a little more than half an inch long, armed with eight longitudinal cutting crests, projecting to the extent of a quarter of a line from the surface. This is carried down upon a mandrin, previously passed through the stricture, and the incision made from before backwards. 2. One called a bridle-cutter, (coupe-bride) resembling the exploring sound of the same surgeon. 3. One more complicated than the other two, consisting of a canula, cleft laterally for about half an inch at its anterior extremity for a sliding semicircular blade, and notched upon the opposite side to the depth of a quarter of an inch, to accommodate the rod which moves the little bar at the end. The instrument with the knife concealed, is carried down to the narrowed part. The oval bar is first pushed on with the rod, and then retracted, so as to hitch against the bridle. A turn is then given to the canula, in order to bring the knife on the same side with the fold, which is to be divided by pressing the blade from before backwards against the bar." Some twenty cases are reported as treated by an instrument similar to this, by Dr. Victor Ivänchich, of Vienna, who has written a learned and practical essay on the subject of strictures, which was published in 1846. It will be seen that all these instruments pre-suppose the stricture *passable.* They are therefore useless in the impassable form of the disease. Even in the ordinary and most common forms of the disease the orifice of the stricture is found on one side or the other, whereas these instruments are designed to pass through a *central* opening. Mercier, in his remarks on the anatomy, pathology and therapeutics of strictures of the urethra, published in the Gazette Medicale of April 5, 1845, has the following words on this subject: "L' orifice du retrécissement quelquefois central se trouve ordinairement plus prés d'un côté que de l'autre, et particulièrement de la parte superieure, à en juger par les empreintes qu' ont fait dessiner Ducamp et M. Segalas. On conçait qui cette paroi, qui est adhérente au corps du pénis obeisse moins qui l'inferieure qui est libre à la force centripète qui opère le retrécissement; mais jè crois aussi avoir remarqué que l'altèration de tissu existe plus souvent et à un degré plus avancé sur la partie inferieure." The difficulties which accompany the use of these comparatively modern instruments for cutting the stricture, have driven surgeons to the old practice of dilatation; caustic, for the most part, as we have seen, having been proscribed by the most distinguished surgeons. Velpeau, as is usual with him when prejudiced against any practice, uses the following strong and emphatic language against cutting strictures: "C'est donc une méthode que ne pourrait convenir qu' aux brides, aux reserrements valvulaires ou en demi-lune, aux nodosités fibreuses, et qui, hors de là, ne peut guère être tentée que par des gens irréfléchis denués de connaissance précise soit en anatomié soit en chirurgie, ou par des specialistes." He then proceeds to state the instruments used, and begins with that of Dorner, who, Ivánchich says, " hat eine Röhre angegeben, durch welche ein stilet mit einer Lansettspitte," &c. He then speaks of Dr. Physick's, which was an instrument " from which a lancet *cutting on its lower edge,* could, by means of a stilet, be projected from the entering end."*

The ingenious instrument of Dr. Chew, made in this city by Mr. Schively, in 1828, consists of an ordinary silver sound, either straight or curved, and split at the entering end, so that an elliptical double edged knife can be projected, for a few lines, by pushing on a button which is attached to the stilet of the instrument. The point of the

* Pancoast's Operative Surgery.

knife is blunt and pierced for the passage of a silver wire. This wire, like that of Amussat and others, is the leader of the knife, and *must* pass the stricture ere the incision can be made—a sad defect, as may be seen by the remarks above on the position of the orifice of the stricture. Yet Prof. Pancoast says: "The probe head of the wire is gradually pushed on separately *through the stricture, which it readily passes* on account of the central position it necessarily occupies in the canal." Whether this "central position" refers to the stilet or the canal, it is equally fatal to the conclusion, for it is known that the orifice is *generally* on one side of the canal. The blunt extremity is also an objection; a free incision cannot be made. Dr. Physick's is objectionable, because it cuts only one side, and that downwards.

Malgaigne, in his Medicine Operatoire, page 668, remarks that, "Les scarifications (ou incisions internes) offrirent sans doute un grand avantage pour la dilatation au canal. Mais aucun des instruments ne nous parait assèz sûr dans son action. Il nous parait qu'au scarificateur pour remplir le but, doit, 1, agir sur tout l'éntendue de l'obstacle, et ne pas aller au-delá; 2, agir par incisions longitudinales; 3, inciser de la base du rétricssement á sonborde libre, pour être sûr de le deviser en entier."

This cannot be done by any of the above instruments; hence, we believe, the want of confidence which the profession has manifested in them. The accompanying cut represents an instrument which we have used with complete success, in the cartilaginous and impermeable form of stricture; in some cases when the popular mode of dilatation had entirely failed—and in fact (which we believe is generally the case in this form of stricture) had only increased the deposit by keeping up the irritation from time to time, in the urethra. We cannot understand, in fact, how dilatation in this form of stricture, can ever effect a permanent cure. It must produce merely a temporary expansion of the parts, unless it establishes ulceration, which of course would tend to destroy both stricture and urethra.

The objections to cutting, offered by Velpeau, that the incisions will heal by the first intention, we think cannot hold, especially if the stricture be completely divided. The urine will certainly act as a foreign and irritating body, and prevent the adhesion of the parts; and we have found it so in practice after the use of the above instrument. We have not found that incision is a mere prelude to more complete dilatation, but would strenuously recommend that no dilatation be resorted to after incision; except merely the introduction, now and then, of a common sized silver sound, as a means of precaution only. To attempt to dilate, in our opinion, would be to bring on additional irritation; and thereby tend to establish the stricture again. The stricture deposit is no doubt kept up by the continual or occasional pressure on the part from within, by instruments, or from without, by the action of the muscles.

The cut represents a flexible catheter,* with a slit in the distal end, perpendicular to the instrument, some two and a half lines long. A ring which is thick and strong, is attached to the proximal extremity. The stilet is two lines in thickness, with a button bur, on one end, which moves on a screw thread, to the extent of a quarter of an inch, and may be in this way moved backwards or forwards so as to control the extent to which the stilet is projected into the catheter. The other end of the stilet, has inserted into it a small blade, projecting three lines beyond the point of the stilet and the extremity of the catheter, which is the shape of the pointed thumb lancet, presenting a sharp edge, cutting *both* ways, upward and downward. The diameter is nearly the diameter of the catheter. The extent, forwards, to which the incision will extend, depends upon the position of the button on the other extremity. Three lines is the extent to which it may be pushed forwards. On account of the flexibility of both stilet and catheter, the instrument is equally adapted to the straight portion of canal as to the curved. Dr. Physick, it will be remembered, used two instruments, one for the straight, and the other for the curved portion of the urethra.

The following case will illustrate the practice with this instrument.

Mr. S. a gentleman about thirty-three years of age, applied to me for advice for a stricture which had been growing about nine years. It had originated in a most violent attack of gonorrhœa while at sea. Shortly after the cure of the gonorrhœa which was a long time in progress, a slight dificulty in urination was perceived about five and a half inches from the mouth of the urethra. This gradually increased, until, about five years ago, it became necessary to apply for medical assistance. Relief was obtained by the introduction of wax bougies and catheters, in the usual manner. The stricture, however, continued to increase, and defied all attempts on the part of several intelligent surgeons at permanent dilatation. So much had it contracted, that there was danger of a total stoppage, which had in fact frequently taken place for hours at a time, subjecting him to great danger of rupture of the bladder or urethra, and the usual results, fistulæ in perineo. When he applied to me he was totally unable to introduce any instrument, however small, as he had

*The size of the instrument is reduced two-thirds, in the drawing.

been in the habit of doing, for the purpose of relieving himself. No attempt on my part was successful in introducing an instrument into the bladder, or through any portion of the stricture, which commenced five and three eighths inches from the orifice of the urethra. In addition to this, being of a highly nervous temperament, the mere attempt at the introduction of an instrument, produced at first, fainting. Spasm of the parts also took place on the least irritation. His general health, in other respects, was good. The sexual function, he informed me, was languid, and the effort at coition ineffectual—the semen passing off some time after the orgasm guttatim; he has been married to a healthy and well formed lady for four years. She has had no children, and has not conceived.

After reflecting upon his case, I began to think that some means should be devised to divide at once this thickened and hardened stricture; for it could be easily felt through the perineum, in the form of a round hard body. I called upon our best surgical instrument makers, but found that they had no instruments for cutting strictures, as they were not used by the profession. After some consultation with Mr. Schively, I got him to finish me the one represented in the cut. With this instrument, on the 9th of September, after measuring the distance of the stricture, oiling my instrument, &c., I cut the stricture. The patient was seated in a chair, the instrument, with the lancet retracted, passed down to the obstruction; was held in the right hand with the index finger upon the top of the burr; the index and other fingers of the left hand were pressed upon the perineum, and served to guide the point of the instrument. When it was properly adjusted, so as to cut in the axis of the urethra, the burr was thoroughly pressed upon by the thumb of the right hand, and made to descend to the end of the catheter, the blade of course projecting its whole extent. The patient did not move a muscle of his face, and after partially withdrawing it, the second incision was made, without pain. The stilet was then retracted, and the whole instrument withdrawn, when, on introducing a gum catheter, it was found that it would pass half an inch further. To be sure of producing no mischief, I directed the patient to call the next day at the same hour.

10th. The patient had suffered no inconvenience from the incision, only one or two drops of blood had passed. In introducing the instrument it could be distinctly felt to pass the hard walls of yesterday's cut, requiring a very little more pressure than the other part. Two incisions were made as before, and a gain was made of nearly three fourths of an inch, making the improvement one and three fourth inches. The pain on cutting was rather more to-day than yesterday, but not enough to induce the patient to move the muscles of his face. Several drops of blood passed after the instrument. The stream of urine is rather better.

11th. One incision to-day with more than half an inch advance; rather more blood than yesterday, but very little pain. Suffers no pain in the interim between the operations. The stream of urine improves daily; very slight smarting after each incision.

13th. The fourth incision was made to-day in the same manner, with the improvement of *introducing* a small catheter into the bladder, the first for two years!! In the passage of this instrument, which was allowed to remain a short time in the canal, it was evident that the difficulty was not yet overcome. The catheter came out of a spiral form, and bent with the point upwards.

The stream of urine improved. More pain during this operation (cutting) than previously, but less blood.

14th. Prepared to use the Letheon, expecting to experience much pain, but on trying the metallic sound I found it would pass very easily, and a voluntary attempt at urinating produced a very good stream. I recommended him to wait a few days to see whether the relief would be permanent. He returned on the 16th, when I found that the sound which had passed so easily before, would not now pass. Some obstruction near the neck of the bladder, though the urine passed very well. I directed him to call on the next day, when I would try a silver catheter.

17th. Neither the sound or silver catheter will pass the obstruction. The parts cut are easily passed, but there seems some impediment still. Operated for the fifth time, by simple incision, when on withdrawing the instrument, I *dropped* the silver catheter into the bladder. About half a drachm of blood followed this incision, and it was more painful than the others, but the passage is apparently complete, and permanently opened. The patient continued to call upon me every few days, and informed me that the silver catheter continued to drop into the bladder. The urine passed as free as ever it had, and not an unpleasant symptom had followed. I directed the occasional use of the silver catheter as a precautionary measure, but I believe it is of no further use. The cure remains complete up to the publishing of this article.

The incisions from without, which are the next form of incisions to be considered, can scarcely, we think, with the exception of *some* cases of fistulæ in perineo, be necessary. The plan of Sir B. Brodie, in his case, in which he operated with a modification of Mr. Strafford's instrument, and in which he *first made an external incision for the purpose of guiding more surely the instrument*, is one which, to say the least of it, as far as the stricture was concerned, was entirely unnecessary. The plan of cutting down upon a sound, and opening the urethra by projecting a trocar-pointed perforator, towards the external orifice of the urethra, is one that is proper in very bad cases of closure of this canal, complicated with one or more fistulæ.

It does not enter into our present plan to speak of the causes and treatment of fistula in perineo, as connected with stricture. We would merely state in conclusion, that our remarks resolve themselves into the following heads.

1stly. That stricture may exist in one of these forms.—Simple, as the result of recent or present inflammation in the part, producing a contraction of the part.—Confirmed, or that in which a deposit of some kind has taken place outside of the lining membrane of the urethra, so as to infringe upon the diameter of the canal. The most persistent, and formerly considered the most incurable of these forms, being that in which the deposit is cartilaginous. —Any one of the above forms may be complicated, and frequently is, with spasmodic stricture, which latter may also exist.—Without any permanent deposit.

2dly. That it is a disease, when found in the permanent form, seldom entirely cured by the ordinary means.

3dly. Some three forms of the permanent stricture may be relieved, and sometimes cured by rupture and dilatation.

4thly. That the more persistent forms of the disease had better be *cut*, whether combined with spasmodic action or not.

5thly. That the merely spasmodic form must be treated 'according to circumstances.'

## Figures and Tables

**Figure f1:**